# Evaluation of the Effectiveness of Dry Needling in the Treatment of Myogenous Temporomandibular Joint Disorders

**DOI:** 10.3390/medicina58020256

**Published:** 2022-02-09

**Authors:** Juan Dib-Zakkour, Javier Flores-Fraile, Javier Montero-Martin, Sara Dib-Zakkour, Ibrahim Dib-Zaitun

**Affiliations:** 1 The Surgery and Odontostomatology Doctoral Program, University of Salamanca, 37007 Salamanca, Spain; juandib97@hotmail.com; 2 Department of Surgery, University of Salamanca, 37007 Salamanca, Spain; javimont@usal.es (J.M.-M.); ibrahimdib@usal.es (I.D.-Z.); 3 Departament of Dentistry, University of Salamanca, 37007 Salamanca, Spain; sdib@usal.es

**Keywords:** temporomandibular dysfunction, deep dry needling trigger points, myofacial pain, randomized clinical trial

## Abstract

*Background and Objectives*: The objective of our clinical trial was to determine the effectiveness of the deep dry needling technique (DDN) (neuromuscular deprogramming) as a first step in the treatment of temporomandibular disorders. *Methods and Materials*: The double-blind randomized clinical trial comprised 36 patients meeting the inclusion criteria who had signed the corresponding informed consent form. The participants were randomly distributed into two groups, the Experimental group (Group E) and the Control group (Group C). Group E received bilateral DDN on the masseter muscle, while Group C received a simulation of the technique (PN). All the participants were evaluated three times: pre-needling, 10 min post-needling, and through a follow-up evaluation after 15 days. These evaluations included, among other tests: pain evaluation using the Visual Analog Scale (VAS) and bilateral muscle palpation with a pressure algometer; evaluation of the opening pattern and range of the mouth, articular sounds and dental occlusion using T-scans; and electromyography, which was used to evaluate the muscle tone of the masseter muscles, in order to control changes in mandibular position. *Results*: Digital control of occlusion using Tec-Scan (digital occlusion analysis) showed a significant reduction both in the time of posterior disclusion and in the time needed to reach maximum force in an MI position after needling the muscle, which demonstrated that there were variations in the static position and the trajectory of the jaw. The symmetry of the arch while opening and closing the mouth was recovered in a centric relation, with an increase in the opening range of the mouth after the procedure. *Conclusions*: facial pain is significantly reduced and is accompanied by a notable reduction in muscle activity after needling its trigger points.

## 1. Background and Objectives

The temporomandibular joint (TMJ), a form of bicondylar diarthrosis that establishes a double connection between the jaw and the temporal bone working symmetrically, is one of the most complex articulations in the body. According to a recent study evaluating historical case series of representative samples of the Spanish population, 12% of adults and elderly patients in Spain experience pain-related symptoms in relation to the muscles of mastication and/or the TMJ [1]. 

At present, the origin of temporomandibular disorders is considered to be multifactorial, and a series of predisposing or precipitating factors has been described in relation to anatomy, occlusion, parafunction, trauma, and psycho-emotional conditions [2]. These disorders are frequently found to be accompanied by tension-type headaches and other neurological episodes (depression, anxiety) [3]. Furthermore, there is evidence of greater prevalence in women [4], which seems to be a significant prognostic factor in all the historical case series evaluated in Spain in the latest national surveys on oral health [1].

Multiple therapeutic strategies have been implemented to alleviate the pain and discomfort associated with musculoskeletal pathology in the orofacial area, with different degrees of invasiveness (splints, medications (infiltration of corticosteroids), physiotherapy, regenerative infiltrations (although though the effectiveness of platelet concentrates is at the center of a recent academic debate, since injections of Plasma Rich in Growth Factors or PRGF modulate the inflammatory response and have regenerative activity in damaged joint components of the TMJ), or surgery) [5,6,7,8,9,10]. Nevertheless, there is still little certainty on the matter. At present, the most consensual approach is to only treat conditions that impact on quality of life through non-invasive means, except when the general repercussions of the clinical presentation worsen, in which case combined and progressively more invasive strategies should be implemented when previous efforts have failed [11].

In this sense, the dry needling technique could be a promising therapy for presentations with chronic muscle involvement (myalgia, referred myofacial pain, or direct myofacial pain) [12]. This minimally invasive technique is based on the insertion of a low-caliber needle, without any additional substances, into myofascial trigger points, which are irritable nodules of a tensed band composed of hypertonic muscle fibers [12]. Two types of dry needling technique exist, based on the depth to which the needle is inserted: superficial needling, or Baldry’s Technique, in which the needle is inserted up to the subcutaneous cellular tissue overlying the myofacial trigger point; and deep needling, in which the needle is inserted into the muscle with the intention of reaching the myofacial trigger point [13]. The process behind this technique is the generation of controlled microspasms in the affected muscle area, which alternate with periods of muscle relaxation, with various studies supporting its therapeutic effectiveness [14,15,16].

In the orofacial area, several authors have studied the effectiveness of dry needling on the muscles of mastication in order to increase the pain threshold to pressure and to maximize the free-of-pain opening of the mouth [17,18]. The study conducted by Luis-Miguel Gonzalez-Perezse et al., focusing on the application of DDN in the lateral pterygoid muscle, which should always be treated under strict ultrasound control due to its complex accessibility and management, reported a reduction in pain and an improvement in maximum mouth opening mobility, jaw protrusion and laterality [18]. This was in agreement with the results obtained in a different DDN study on the temporal and masseter muscles, where the effects measured immediately after and a week after the procedure were evaluated [19]. However, other authors attributed these effects to placebo, although their small sample size (*n* = 10 per group) could have limited their statistical inference capacity [20,21].

Our study offers an affordable, swift, and easily applicable alternative, which is useful not only as an isolated treatment for muscle dysfunction, but to facilitate or enable the use of other types of intervention carried out by odontologists, such as the reversion to the baseline physiological position of the jaw in centric relation (CR).

The purpose of this study was to evaluate DDN’s effectiveness in the treatment of myogenous forms of temporomandibular joint disorder by monitoring the activity of the masseter muscle, bite force, mouth opening range and symmetry, as well as changes in the position of the jaw after applying DDN.

## 2. Materials and Methods

The sample size necessary to undertake our study was calculated with 95% confidence and 80% statistical power.

We conducted a randomized double-blind clinical trial with a total sample size of 36 patients. Subjects in the sample were recruited from patients attending the Clinical Odontology Consultation of the Faculty of Medicine of the University of Salamanca, as well as university students and other external individuals who wished to participate and met the following inclusion criteria: subjects between the ages of 18 and 40 years, with myofacial pain due to temporomandibular dysfunction at the time, diagnosed using the Research Diagnostic Criteria (RDC) [21], who had teeth or carried partial fixed prostheses, and showed signs of temporomandibular articular pathology (without determining the degree of TMJ involvement). Patients had not received any other form of treatment related to TMJ disorders.

The exclusion criteria were: a confirmed or suspected diagnosis of an inflammatory disorder (arthralgia), the presence of an oral or dental infection, a confirmed or suspected diagnosis of neurological disorders, a history of physical trauma in the head or face, intake of anticoagulants or drugs for circulation disorders, allergies to metals, and patients with cognitive and/or communication impairments that could hamper necessary data collection [22]. 

At the beginning of the study, and in order to verify the inclusion criteria, all participants completed a self-administered questionnaire (ANNEX 1) based on the guidelines of the Research Diagnostic Criteria (RDC) for temporomandibular disorders [23].

### 2.1. Intervention

Each patient was asked to fill in a specific questionnaire on the level of pain suffered before starting the procedure. Pain was evaluated using a visual analog scale, thus allowing the patients to express their own sensation of pain.

The sample was divided into two groups:

Group E: intervention group, who received the deep dry needling technique.

Group C: placebo group, on whom deep dry needling was not performed.

Next, digital occlusal registration was carried out using a T-scan to determine the occlusal contact points, the percentage of occlusal force applied on each tooth, the time needed to reach maximum occlusal force, and the time of posterior disclusion, parameters directly associated with the conditions of the muscles, and which determine postural characteristics at the same time. The electrical activity was registered at baseline and at the maximum intercuspation position (MI) in the masseter muscle after placing the corresponding electrodes in the mandibular angle in parallel to the fibers of the masseter muscle on both sides.

Group E received the DDN treatment in both masseter muscles, using 0.30 × 0.30 mm AGUPUNT acupuncture needles with guides. The technique requires the patient to rest in a supine position, with their eyes closed and with the head rotated towards the right when treating the left masseter muscle and towards the left for the right masseter muscle. The trigger point is identified and marked with a dermographic pen (pain evaluation was carried out through the palpation of different points according to RDC guidelines, using an algometer to determine the exact amount of exerted pressure (ANNEX 3). 

**Figure 1:**
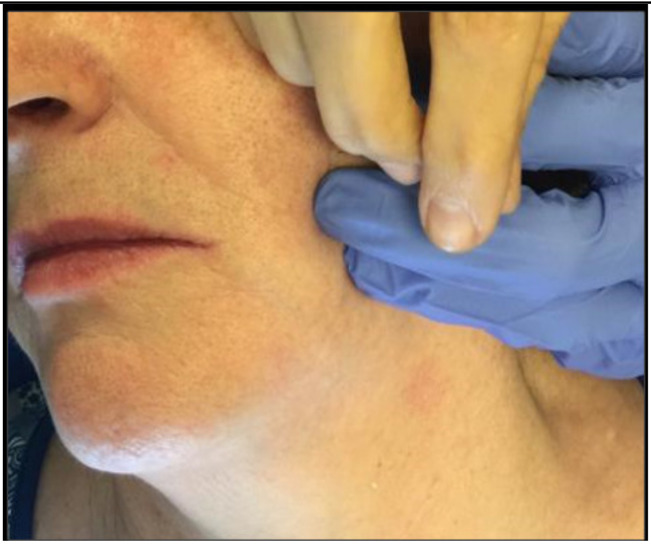
Patient lying in the required position for needling.

The skin was then cleaned and the electromyographic electrodes are placed at the origin and the insertion of the masseter muscle. An antiseptic was subsequently applied on the needling area and the needle was inserted under the aseptic conditions required for the technique. During needling, the physical response of the patient was observed at all times, with the objective of controlling local spasm responses in each masseter muscle Figure 1.

**Figure 2:**
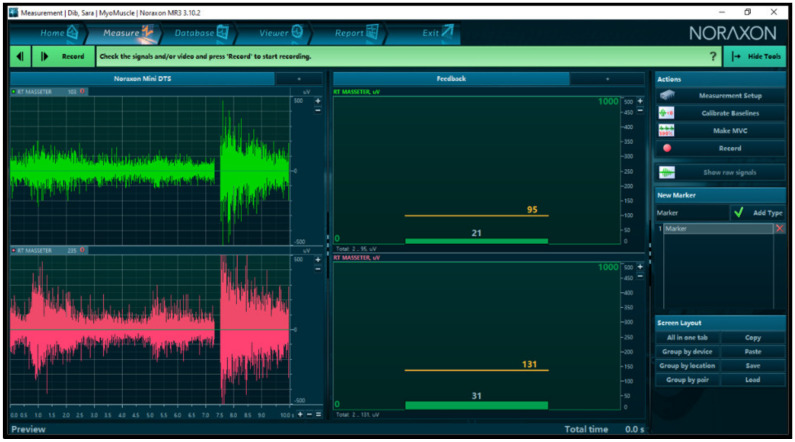
Electromyography surface electrode positioning.

**Figure 3:**
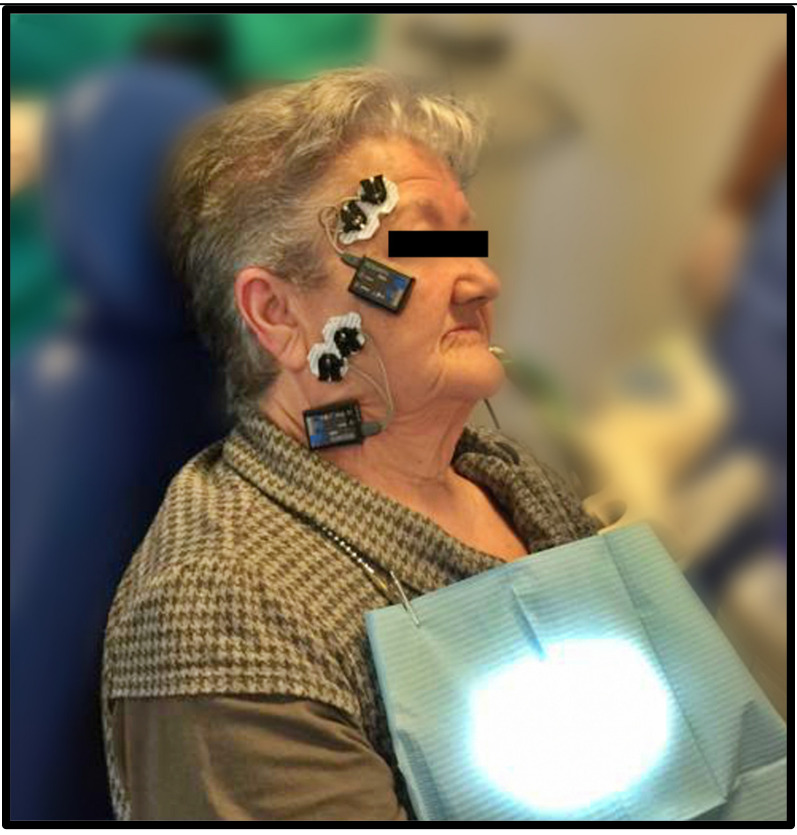
Pre-needling registry of the activity in MI position.

Next, the opening pattern and the measurements of the opening of the mouth were evaluated using a digital caliper, and the joint was auscultated on both sides during the opening and closing movements to assess the characteristics of potential sounds (such as clicks, pops, and so on, as well as their volume and frequency) (Figure 2 and Figure 3).

Ten minutes after the procedure, a new evaluation was conducted (post-needling), which included all the above-mentioned change variables.

**Figure 4:**
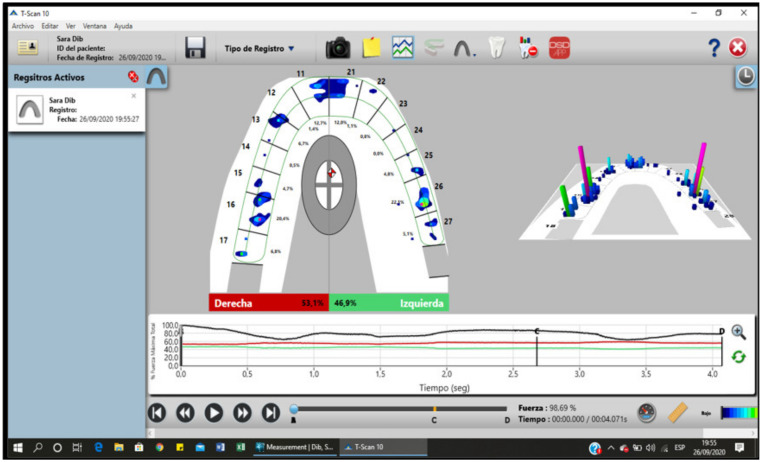
Digital occlusal analysis determining the characteristics of the jaw position.

Group C received a simulation of the DDN treatment. The patient was set in the same position, using the same needle and guide with the same characteristics, and for the same time as estimated for group E, although in this case the safety piece was not removed so as not to needle the patient. All the aforementioned records were obtained out for muscle activity, mandibular position, mandibular opening and closing patterns, and joint noises, both before and after the simulation of the dry needling maneuver (Figure 4).

After conducting the technique, the patient was left to rest for 10 min for the needling to take effect, and afterwards the same evaluation registrations described above were repeated, evaluating the post-needling pain levels. The patient was then asked to fill in the same pain level questionnaire completed at the beginning.

Subsequently, 15 days after the procedure, a new evaluation (follow-up evaluation) was conducted, including, in addition to the change variables registered during the post-needling evaluation, a new, self-administered questionnaire with aspects related to pain and functional activity.

### 2.2. Data Analysis

The data were analyzed using the IBM.SPSS Statistics program, version 23 and were presented with a 95% confidence level. 

## 3. Results

Table 1 offers the variations of the masseter muscle activity values in both sides, comparing electric activity results during the pre-needling and post-needling phases.

The activity of the same muscle in its maximum intercuspation position was also registered following the same protocol, finding changes in the muscle’s electric activity though without statistical significance. These were probably due to the clinical trial’s sample size. We registered the following values: 130.73 µV in the right masseter and 125.55 µV in the left masseter pre-needling; and 112.86 µV and 90.71 µV post-needling. The reduction in activity is notable, especially in the left side. This was probably due to prematurity and interferences in the right side, which were evidenced after analyzing the T-scan results. This situation was no longer noticeable in the different CR positions, where the occlusal irregularities that can affect the electric activity of the muscle were nullified.

**Figure 5:**
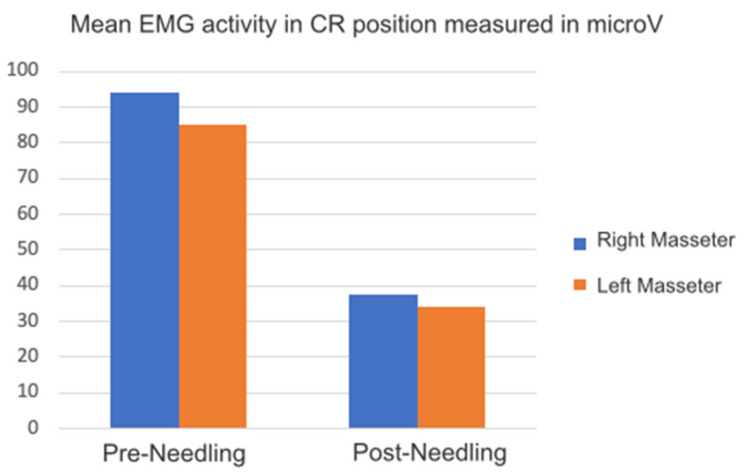
Graph of EMG activity in CR position compared with other techniques (CR-P, CR-L), Table 1.

According to the protocol, regarding the EMG values registered in centric relation positions with different techniques, we found a significant reduction in muscle activity between pre- and post-needling, of > 0.05. Dawson’s bimanual technique CR was associated with the highest reduction percentage of the activity of the right 94 µV and left 85 µV muscles before, as well as 31.71 µV right and 34.14 µV left after needling (Figure 5). This striking reduction, corresponding to CR measurement using jaw induction after achieving a relaxed state, is the only technique that does not entail any dental contact, a situation that favors an increase in muscle activity. 

Considering the EMG, we can sort the methods used for measuring centric relation according to the masseter muscle’s activity based on the reduction in EMG activity between the pre- and post-needling states in the following order: 

CR according to Dawson’s bimanual technique.

CR-P, where centric relation is determined by the closing arch of the jaw up to the first dental contact, with a mean pre-needling activity of 36.91 µV in the right/33 µV in the left masseter and of 24.4 µV in the right/20 µV in the left masseter post-needling. 

CR-L, where the centric relation is measured with Long’s laminae with a mean pre-needling activity of 42.91 µV in the right/36.91 µV in the left masseter and of 30.5 µV in the right/28.3 µV in the left masseters post-needling.

**Figure 6:**
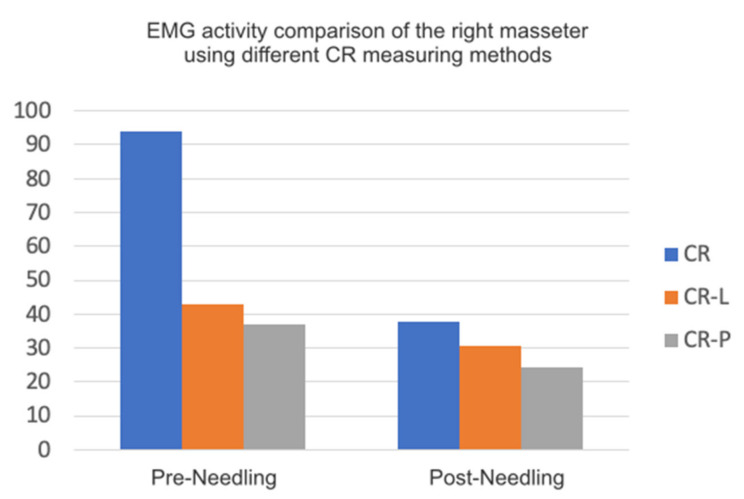
Graph EMG activity comparison of the right masseter using different CR measuring methods. Regarding the opening of the mouth and its symmetry, we found a significant change, of >0.05, in the opening, with a mean value of 44.92 mm pre-needling, measured between the incisal borders of the superior and inferior centrals.

Figure 6 shows the comparison between the three methods of determining the central relation.

**Figure 7:**
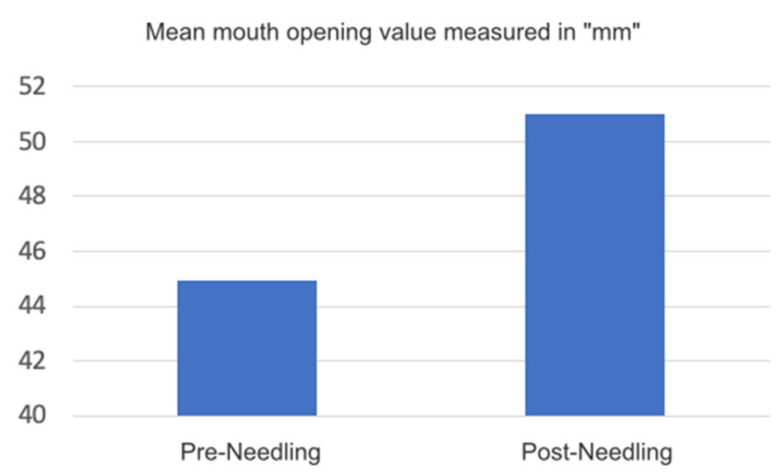
Graph mean mouth opening value measured in mm.

The mean post-needling distance is 51 mm. Figure 7.

For the symmetry of the arch when opening and closing, we registered significant changes. In certain situations, the symmetry was affected by intra-articular morphological and structural changes. These modifications where mostly motivated by masseter muscle relaxation after trigger point needling (Table 2).

**Figure 8:**
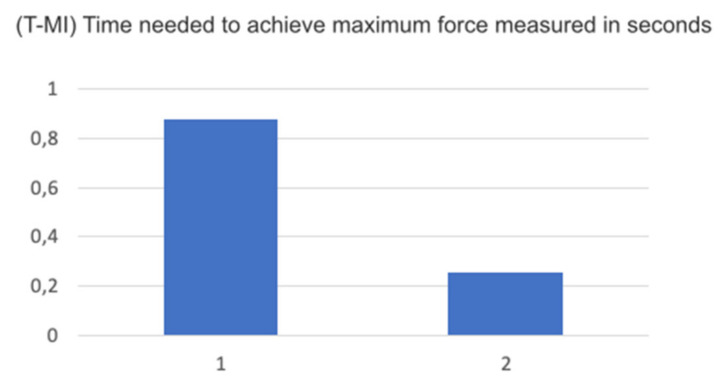
Graph T-Mi, time needed to achieve maximum force measured in seconds.

For the variable of the time needed to achieve maximum force in a maximum intercuspation position, disregarding the determining factor of the muscle due to the needling technique, and measuring using the T-scan, we found a considerable reduction in the amount of time needed, possibly due to the postural change of the jaw after the procedure, avoiding premature reactions secondary to muscle issues (Figure 8).

**Figure 9:**
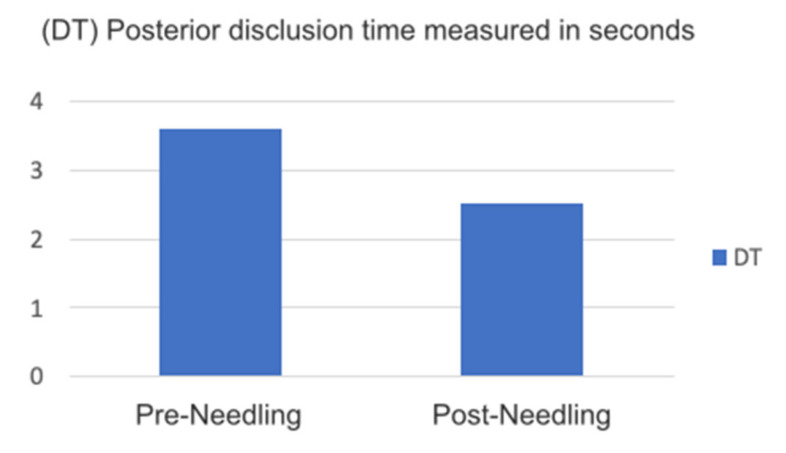
Graph DT posterior disclusion time measured in seconds.

The posterior disclusion time, a major characteristic of optimal functional occlusion, is usually affected in certain circumstances by the situation of the muscle and vice versa, since delayed posterior disclusion is one of the main reasons for increases in facial pain and EMG activity. According to our results, the reduction in DT was significant: >0.05 (Figure 9).

Regarding both articular and facial pain, we registered significant mean values, especially for facial pain, a variable that is directly subject to the state of the muscle. These results are shown in Table 3.

Compared with the results on articular pain measured through VAS in both variables, we observed a smaller reduction in articular pain between the pre- and post-needling states, though intra-articular factors; changes affected this variable more than the effects of the muscle itself.

**Figure 10:**
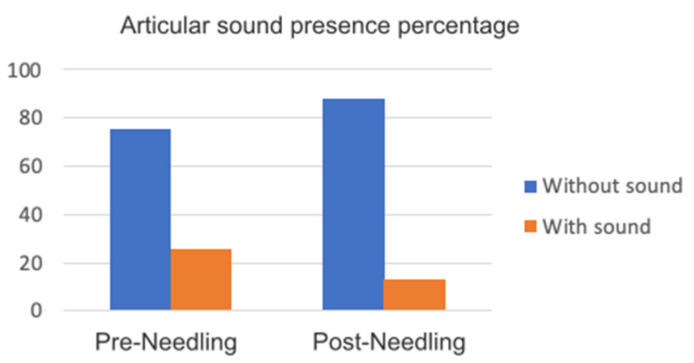
Graph articular sound presence percentage.

The articular sounds showed a noticeable improvement at 10 min after needling of the masseter muscle, considering that the muscle factor plays an important role in adjusting the trajectory that the jaw follows in its movement. The results can be found in Figure 10.

## 4. Discussion

At present, the effectiveness of DDN is more than evident thanks to numerous research studies that have highlighted the importance of ischemic compression and eccentric load in muscle exercises following DDN [21,22].

The first use of DDN therapy was in the 1940s and, since then, numerous studies have tried to analyze this technique in all its diversity. Two meta-analyses comparing the effects of dry needling with wet needling using lidocaine concluded that short-term results are similar [23,24,25,26]. In 2016, the Canadian Agency for Drugs and Technologies in Health accepted the use of DDN in the public health system for the treatment of different musculoskeletal pain syndromes [27,28,29,30]. A recent meta-analysis study on the use of DDN in temporomandibular disorders concluded that the technique in question considerably reduces pain intensity compared with sham therapy [31,32]. In their meta-analysis, Hall et al. concluded that DDN effectiveness is low at the muscular level, but is higher at the neurological level [33,34].

Gatte et al. proved that DDN effectiveness was low-to-moderate in relation to physiotherapy treatment for musculoskeletal pain in the short- or medium-term [35,36,37,38,39]. Gerwin and Shah proved that DDN is capable of interrupting dysfunction at the terminal of the motor end plate, increasing muscle length and reducing the superposition of actin and myosin fibers [40,41,42]. Lui Q.G. reports that DDN helps reduce the range and electric frequency at the terminal of the motor end plate, reducing acetylcholine levels [43,44]. Chou and Hsieh report that the spontaneous reduction in electric activity is associated with a cascade of muscle contractions during the DDN procedure [45,46]. This phenomenon leads to a reduction in acetylcholine levels, causing an increase in t blood flow and, in turn, in local oxygenation levels, leading to muscle relaxation in the area [47]. 

Butts reports that, from a neurophysiological standpoint, DDN reduces both peripheral and central sensitivity by neutralizing nociceptors in the area, modeling de activity of the dorsal spine through the inhibition of the activity of central pain pathways [48,49]. In their studies, Shah et al proved that with DDN, there is an immediate concentration in the area of neurotransmitters, such as calcitonin, as well as of various cytokines and interleukins, both outside and in cellular fluids [50,51]. Hsieh et al. confirmed that DDN models chemical mediators associated with pain and inflammation by increasing B-endorphins [52,53]. It is evident that DDN cannot be the only therapeutic option when treating chronic pain but must be accompanied by other therapeutic techniques [54,55], such as physical exercise, psychological treatment, and the treatment of sleep disorders [56,57]. Woolf considers that afferent signals and their transmission pathways, as well as nociceptor sensors, constitute the most frequent etiology for myofacial pain [58,59,60]. Fernández de la Peña reports that trigger points can be considered peripheral sensors for nociception that contribute to pain propagation [61,62]. This theory suggests that the interactions are bi-directional. Their conclusions concur with our results regarding trigger point needling, which deactivates nociceptors, deprograms the affected muscles, and allows mandibular movements free of muscle conditioning [63,64,65,66].

Clinically, in situations of chronic pain, a proper understanding by the patient of the mechanism behind the pain is considered crucial [63,64,65]. Secondly, understanding the role of trigger point needling, its interventions in nociceptors, their pathways, and the effects of the technique on pain relief are also important [66]. Thirdly, the combination of DDN with a good understanding of the mechanism behind its effects is key in reducing kinesiophobia [67,68].

From our point of view, and in light of the results of our study and the duration of DDN effectiveness, we consider the use of the technique as a neuromuscular de-programmer as the first step in the multidisciplinary therapeutic process of myofacial pain.

## 5. Conclusions

Although, at present, there is no consensus on the effects of DDN, various studies consider that this technique offers swift pain relief despite the short duration of its effects. We observed:

- A significant reduction in facial pain and a reduction in muscle activity after needling trigger points.
- A significant variation in the static position and in the trajectory of the movement of the jaw, determined through digital occlusion control using Tec-Scan (occlusal digital analysis).
- A reduction in the asymmetry of the arch when opening and closing the mouth in the centric relation with an increase in the maximum mouth opening after needling.

## Figures and Tables

**Table 1:** Mean muscle activity value comparison in microvolts (µV) of the masseter muscle in different jaw positions, where RM is relaxed mandible, MI is maximum intercuspation position, CR is jaw in centric relation according to Dawson’s bimanual manipulation, CR-L is centric relation measured using Long’s laminae, CR-P is centric relation obtained in the position of the first dental contact, DT is jaw disclusion time, P is jaw protrusion position and T-MI is time needed to reach the position of maximum intercuspation. We were able to clearly observe a reduction in activity in the right (19 µV) and left (20.36 µV) masseter muscles in a relaxed jaw position before needling the trigger points. After waiting 10 min post-needling and repeating the same measurement, we obtained values of 23.71 µV/17.65 µV, respectively.

	Mean Pre-		Deviation Pre-		Mean Post-				Deviation Post-			
	Right	Left	Right	Left	Cont.		Interv.		Cont.		Interv.	
					Right	Left	Right	Left	Right	Left	Right	Left
RM	19	20.36	19.31	25.05	21	15.8	23.71	17.65	20.06	12.76	40.27	18.04
MI	130.73	125.55	227.83	248.22	229	228.75	112.86	90.71	402.1	402.25	222.34	168.37
CR	94	85	73.5	68.38	94	70.50	37.71	34.14	150.70	106.34	58.71	49.14
CR-L	42.91	36.91	66.96	53.86	92	70	30.50	28.3	64.3	52.5	35.3	30.45
CR-P	36.91	33	54.83	46.77	35	30	24.4	20	50.30	40.23	45.5	35.45
DT	3.59		1.74		3.45		2.09		0.5		1.3	
P	94.82	81	147.46	160.41	174.50	98.75	64.57	51.43	255.03	147.54	123.92	87.74
T-MI	0.87		1.21		0.25				0.6			

**Table 2:** Percentage symmetry of opening and closing mouth.

Presence Percentage	Asymmetry	Symmetry
Pre-	75%	25%
Post-	37.5%	62.5%

**Table 3:** Articular and facial pain.

		Mean Pre-		Dev. Pre-		Mean Post-		Dev. Post-	
		Cont.	Interv.	Cont.	Interv.	Cont.	Interv.	Cont.	Interv.
Mouth Opening		44.92		7.36		51.75	51	2.36	2.64
Facial Pain		7.75	8.57	0.95	0.97	0.5	1.5	0.57	0.97
Articular Sound	No	75%	75%			100%	87.5%		
	Yes	25%	25%			0	12.5%		
TMJ pain		2.50	3	1.73	2.77	1	2.63	2	3.24

## Data Availability

Not applicable.

## Abbreviations

CR	Central relationship
CR-L	Central Relationship through long sheets
CR-P	Central Relationship through the first point of occlusal contact
DDN	Deep dry puncture
DT	Mandibular disclusion time
EMG	Electro-miography
EVA	Visual Analogue scale
MI	Maximum intercuspidation
P	Protrusive position
PP	Placebo puncture
RDC	Research diagnostic criteria
RM	Mandibular rest
TMJ	Temporomandibular joint
T-MI	Time to reach maximum occlusive force
Uv	Micro-volt
